# Supplementary material for: Neck region-microtubule interactions direct counterclockwise stepping of kinesin-1
Source: Biophys J. 2026 Mar 25;125(16):4357–68. doi: 10.1016/j.bpj.2026.03.043 (PMC13507478; doi:10.1016/j.bpj.2026.03.043)
Supplement: Supplementary file 1 [file mmc1.pdf]

Biophysical Journal, Volume *125*

Supplemental information

Neck region-microtubule interactions direct counterclockwise stepping of kinesin-1

Song-Ho Chong and Ryota Iino

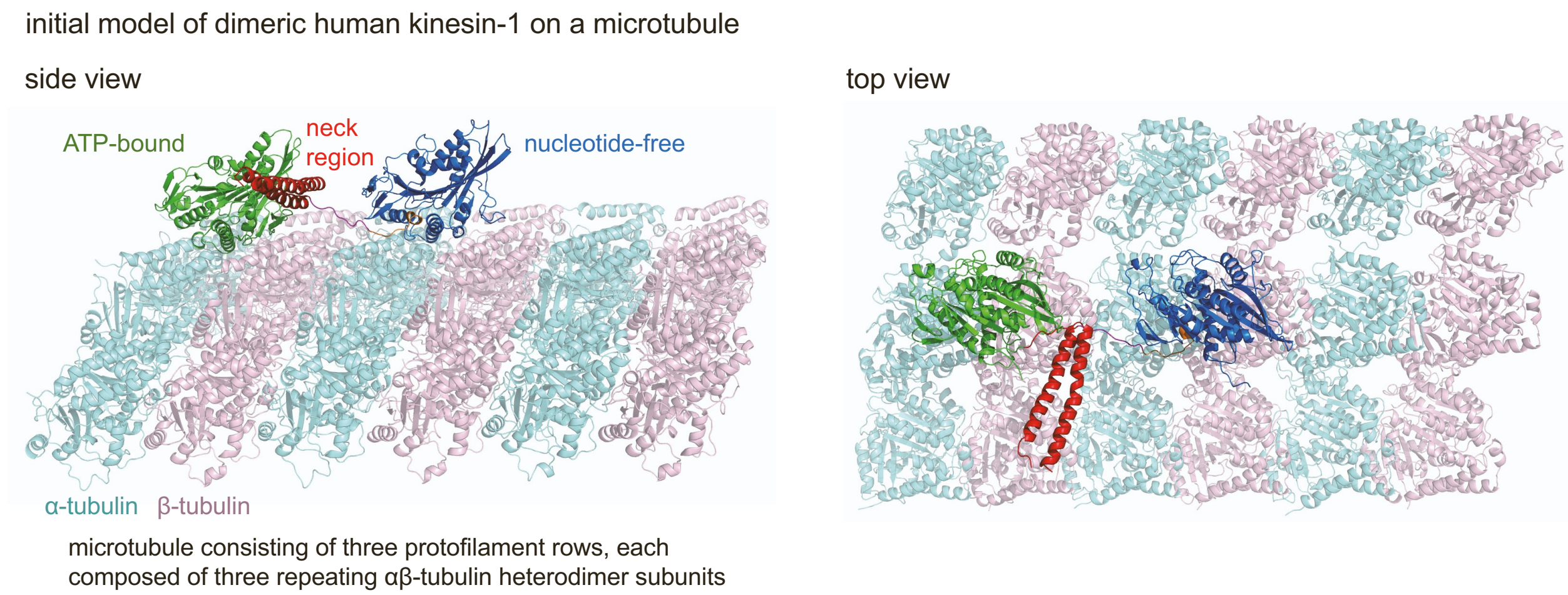

Figure S1: Initial model of dimeric human kinesin-1 bound to a microtubule, shown in side view (left) and top view (right). The modeled neck region is highlighted in red.

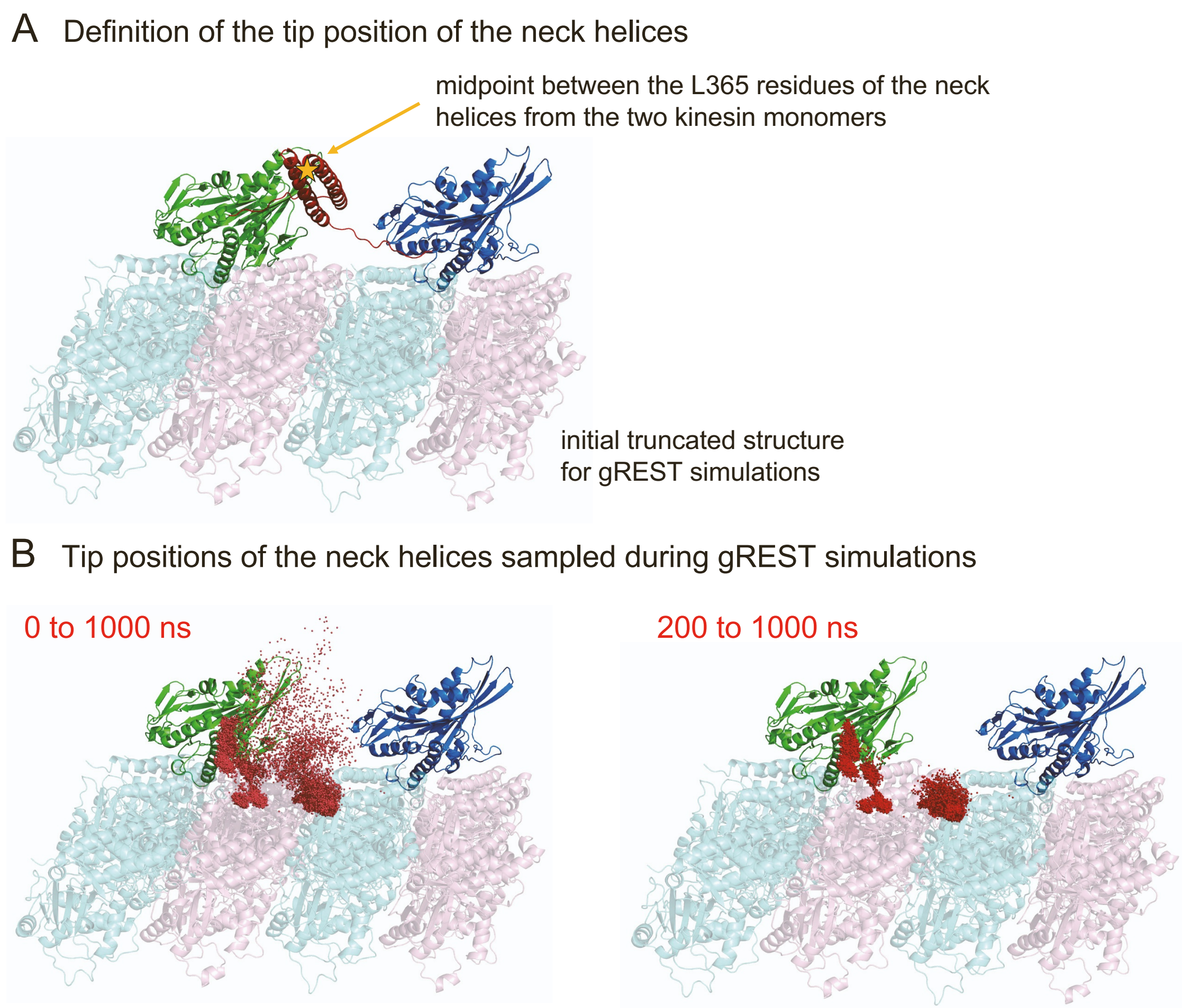

Figure S2: Tip positions of the neck helices sampled during gREST simulations. (A) The tip position for each kinesin dimer conformation is defined as the midpoint between the L365 residues of the neck helices from the two kinesin monomers. The orange star indicates this position for the initial truncated structure used in the gREST simulations. (B) Tip positions sampled over the full course of the gREST simulations (0–1000 ns) and from the later stage (200–1000 ns), excluding the initial 200 ns, are shown as red dots in the left and right panels, respectively.

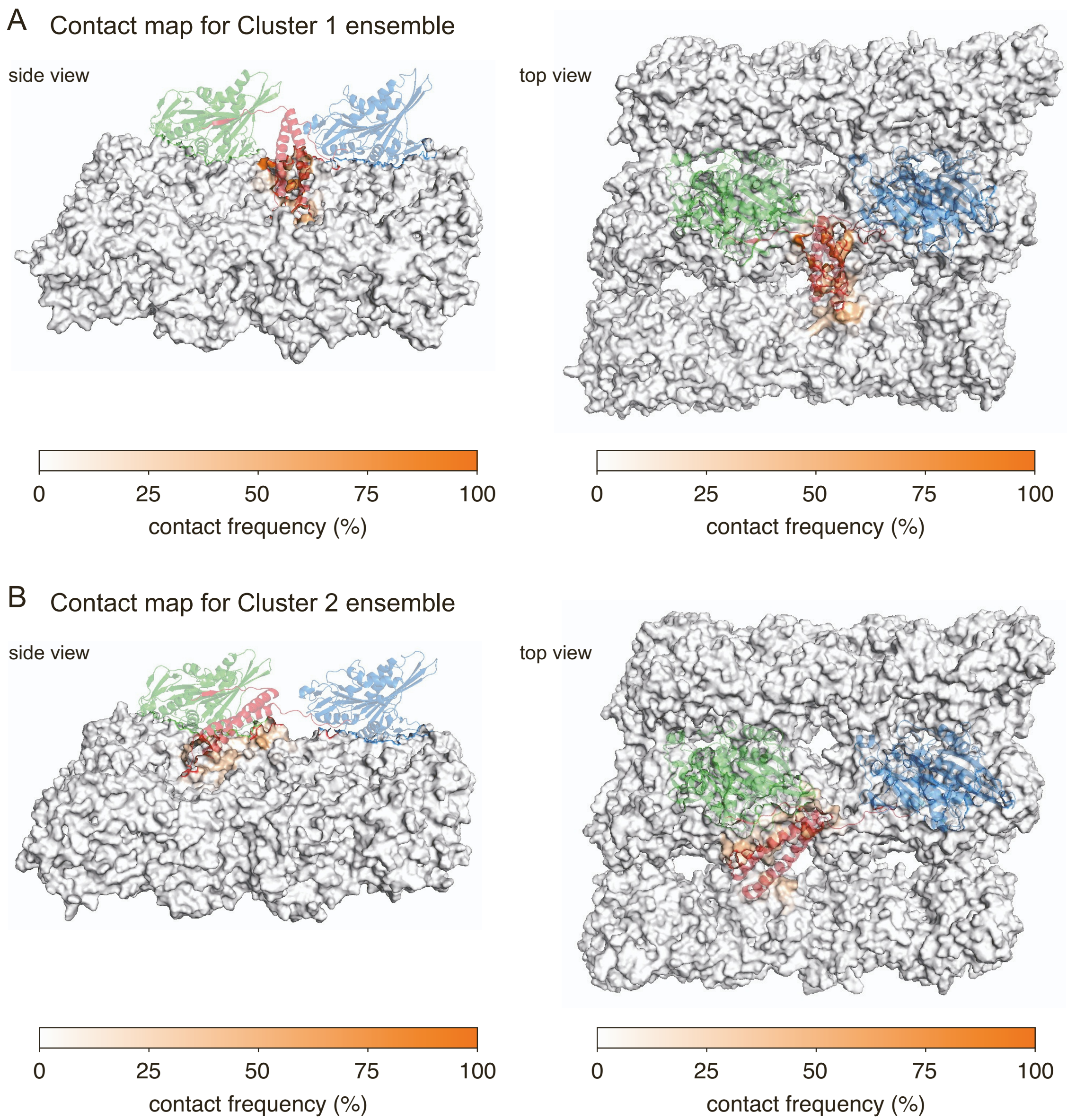

Figure S3: (A, B) Contact maps visualized on the microtubule surface for Cluster 1 (A) and Cluster 2 (B) center structures. The kinesin dimer is displayed in a semi-transparent cartoon representation, whereas the microtubule is shown in surface representation and colored according to the contact frequencies between the kinesin neck coiled-coil and the microtubule surface. Color scale corresponds to contact frequency values ranging from white (0%) to orange (100%). The specific residue-pair contact frequencies are listed in Table S1.

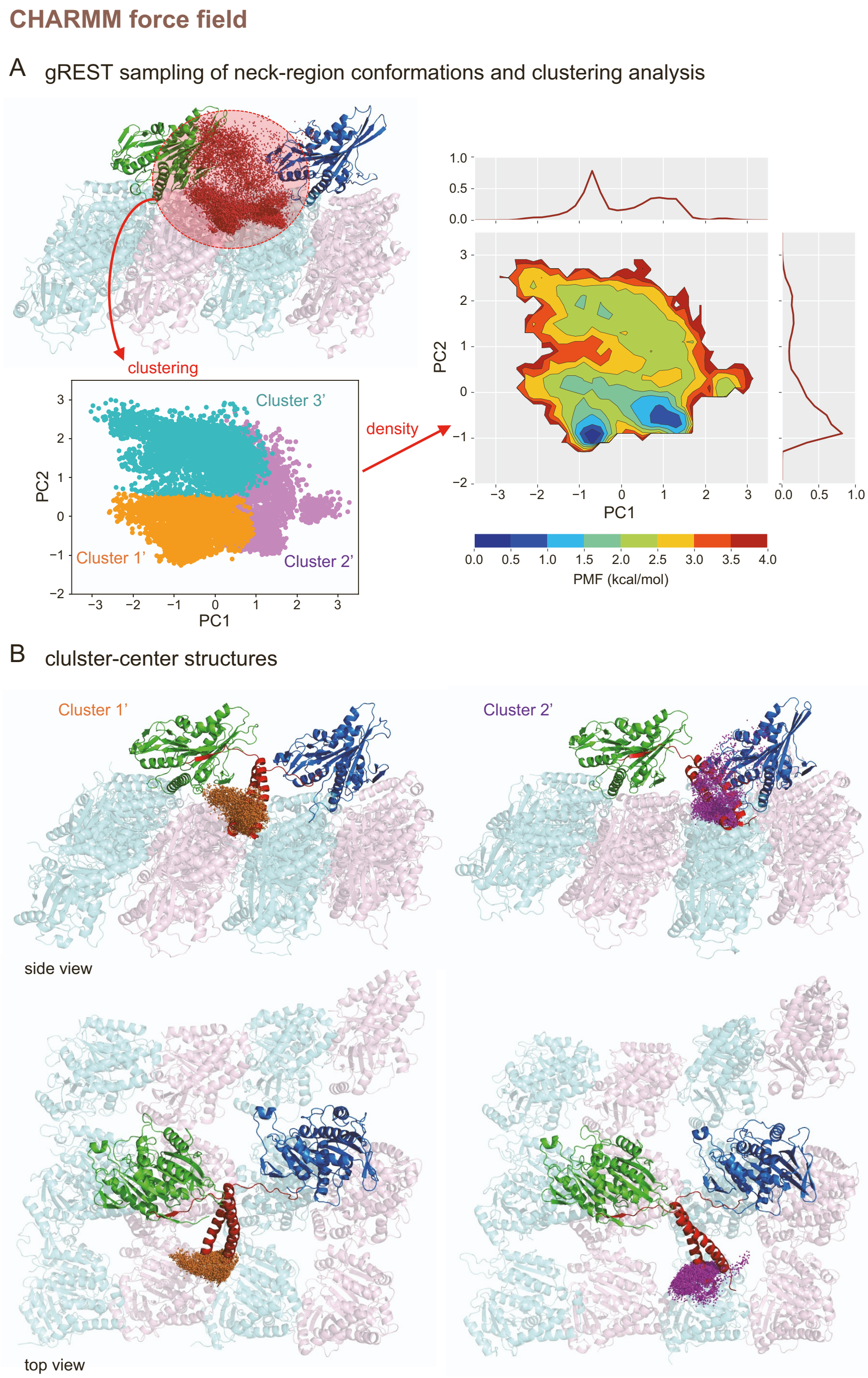

Figure S4: Clustering analysis of neck-region conformations sampled by gREST simulations using the CHARMM36m force field. The same analysis protocol as in Fig. 3 of the main text (which used the AMBER ff99SB-ILDN force field) was applied. Two major clusters were identified and labeled as Cluster 1' and Cluster 2' to distinguish them from those presented in the main text. Although an additional cluster (Cluster 3') can be inferred from the clustering results on the principal component (PC) axes, it is not discussed further due to its low population density. The representative structure of the most populated Cluster 1' closely resembles that of Cluster 1 in Fig. 3B, highlighting the consistency of the neck-region conformation across different force fields.

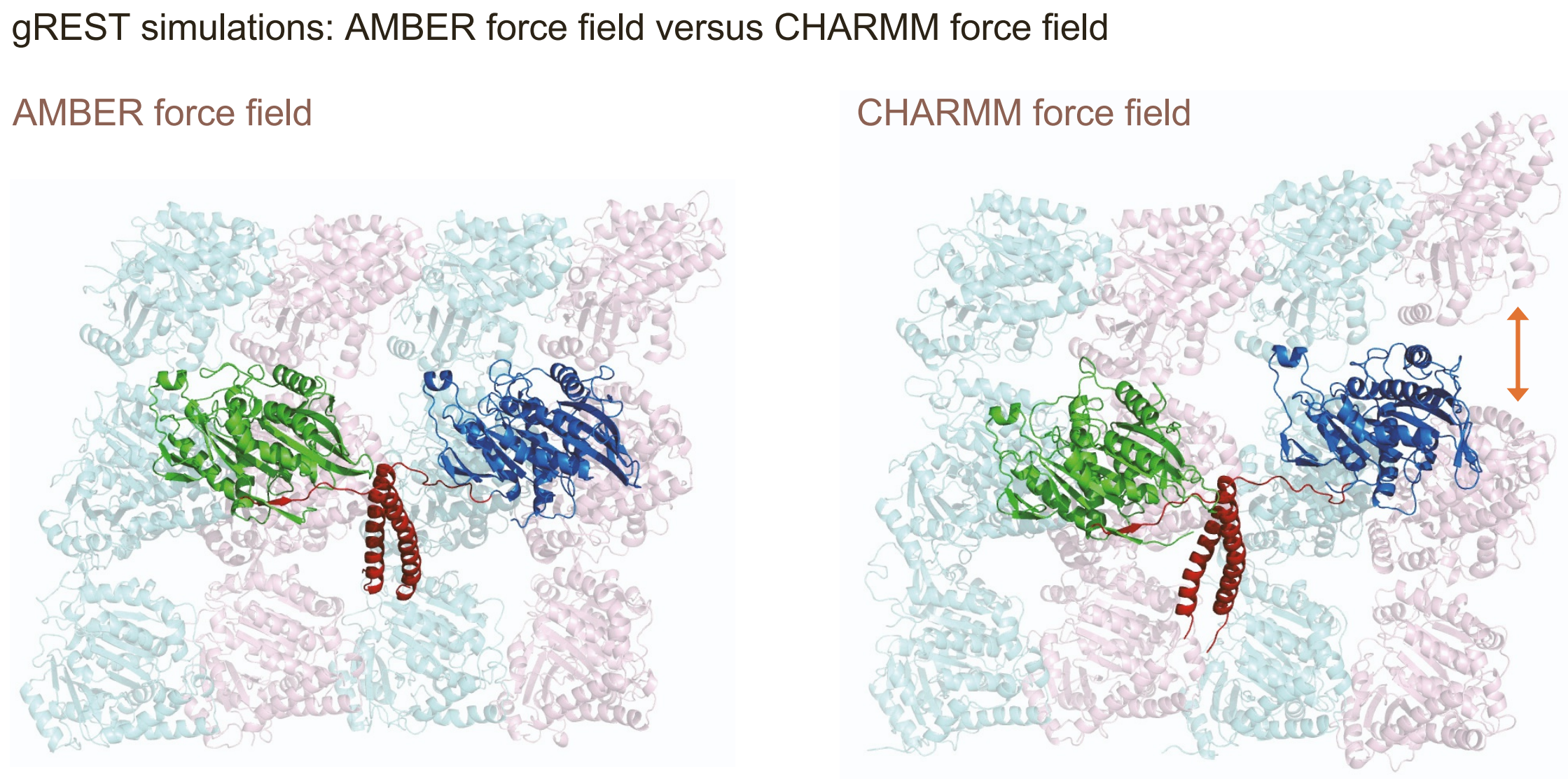

Figure S5: gREST simulations: AMBER force field versus CHARMM force field. Comparison of microtubule structural stability during gREST simulations performed with the AMBER ff99SB-ILDN (left) and CHARMM36m (right) force fields. Each panel shows a representative snapshot from the respective simulation. The AMBER simulation preserved the structural integrity of the multi-protofilament microtubule throughout. By contrast, in the CHARMM simulation, the distance between the central protofilament and one of its neighbors increased markedly, leading to partial disruption of the microtubule lattice.

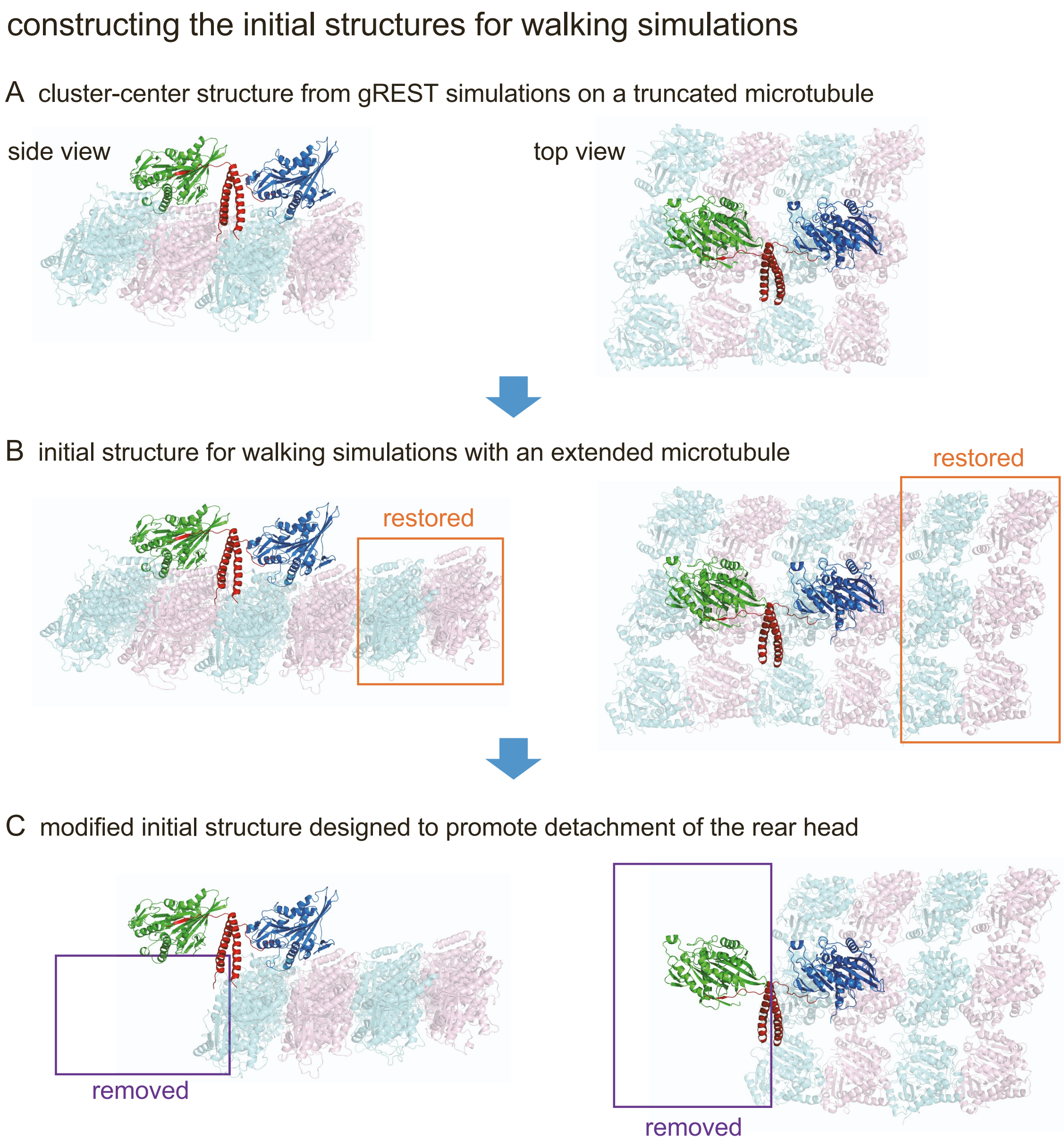

Figure S6: Constructing the initial structures for walking simulations on the microtubule. (A) Cluster 1 structure obtained from the gREST simulations, shown in side and top views, employing a truncated microtubule model. (B) Initial structure for walking simulations with an extended microtubule, in which the distal $\alpha\beta$-tubulin heterodimer subunits removed during the gREST simulations are restored to provide a landing site for stepping. (C) Modified initial structure designed to promote detachment of the rear head, in which the $\alpha\beta$-tubulin heterodimer directly beneath the rear head, along with adjacent subunits from neighboring protofilaments, has been removed.

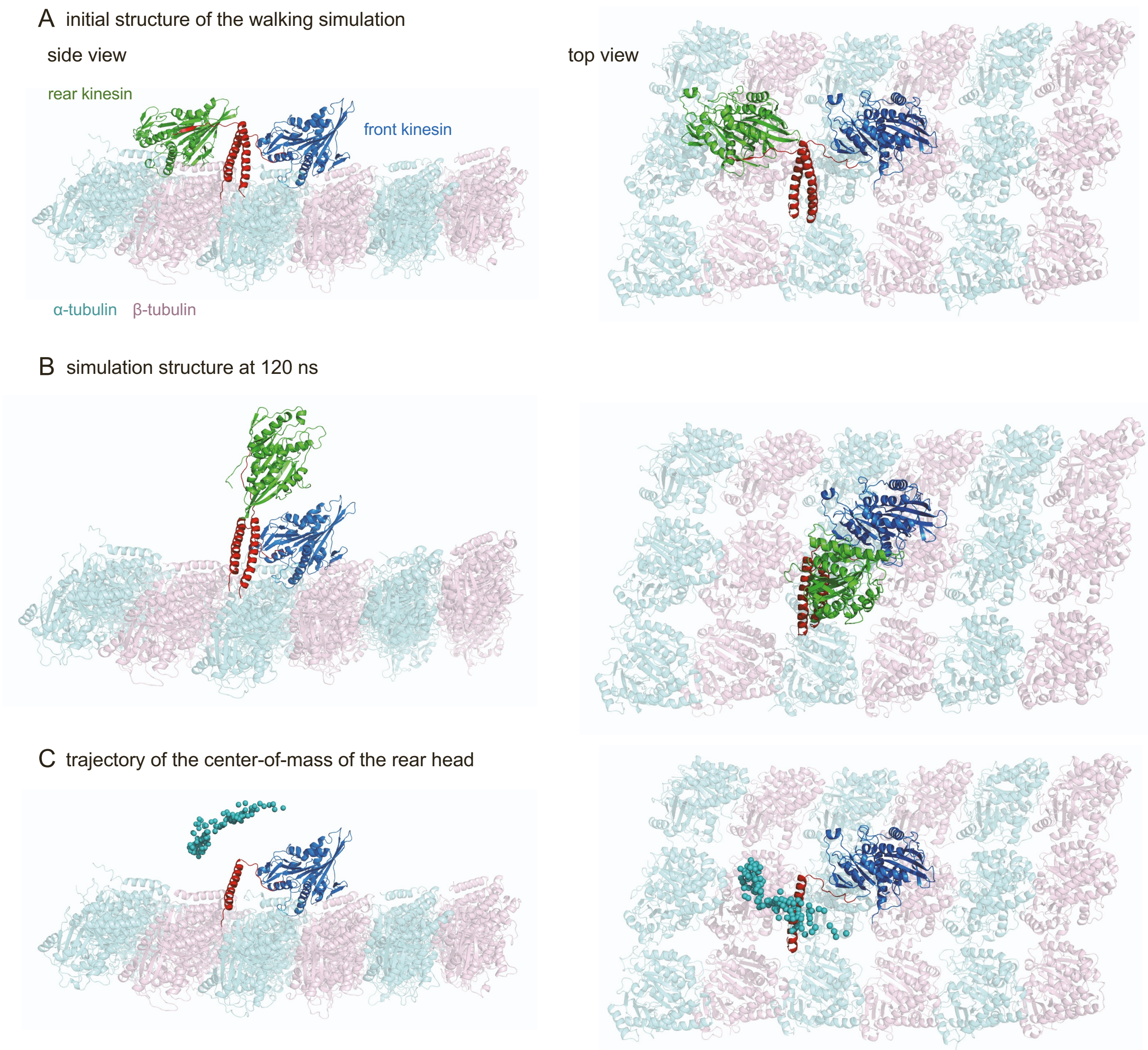

Figure S7: Another representative trajectory of the initial stepping motion of the kinesin dimer on the microtubule. (A, B) Structural snapshots at 0 ns and 120 ns, respectively, from a different simulation starting from the same Cluster 1 conformation. The removed $\alpha\beta$-tubulin subunits have been reintroduced for visualization purposes. (C) Center-of-mass positions of the rear head, sampled every 1 ns from 1 to 120 ns, are shown as cyan spheres to depict the stepping trajectory.

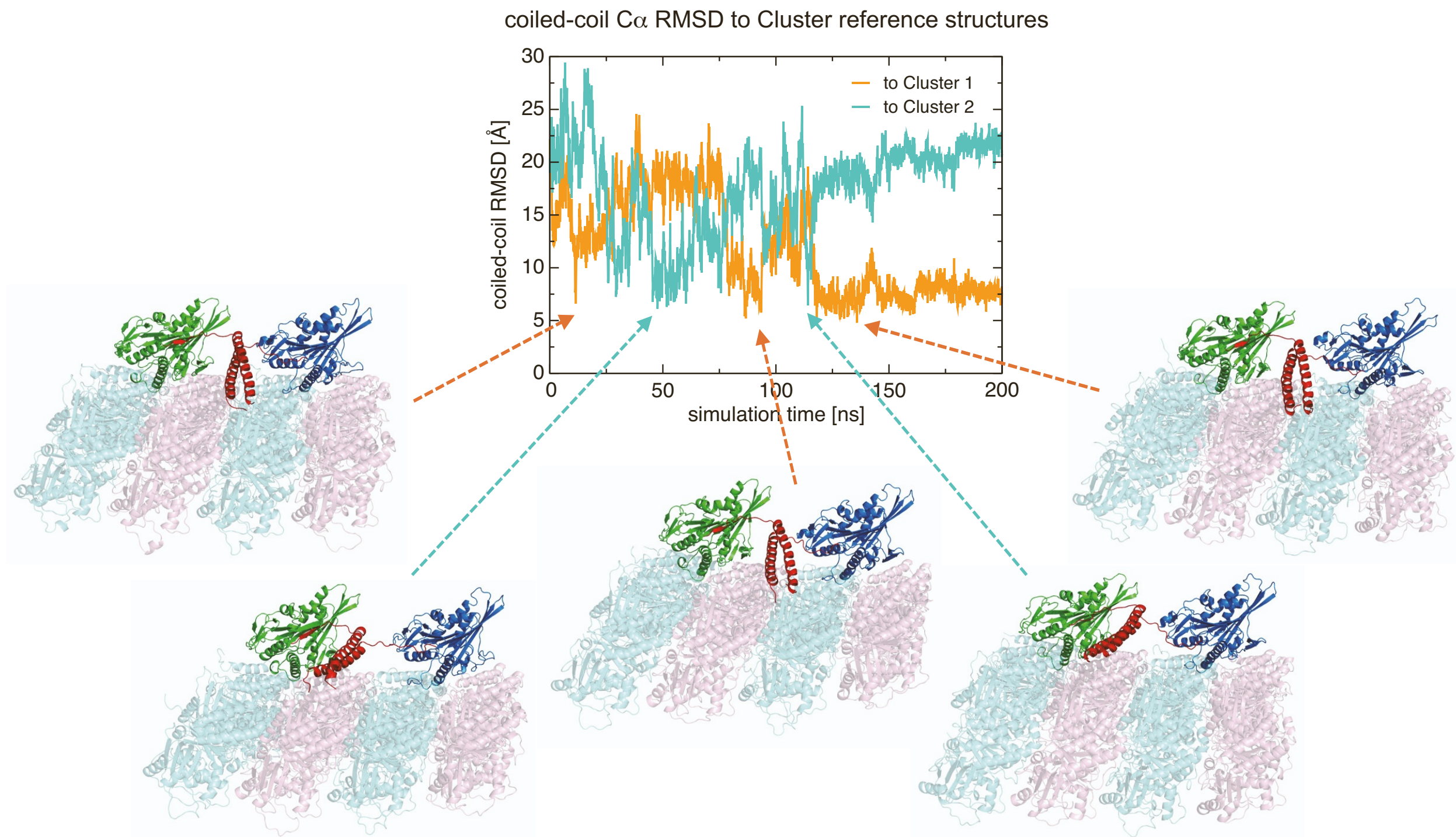

Figure S8: Time series of Cα RMSD of the neck coiled-coil region relative to the Cluster 1 and Cluster 2 reference structures. For a representative gREST replica, simulation snapshots were first aligned using the Cα atomic coordinates of both kinesin heads (residues 1–320) to remove overall translational and rotational motions. The Cα RMSD of the neck coiled-coil region (residues 337–368 of both the front and rear kinesin) was then computed with respect to the Cluster 1 and Cluster 2 representative structures. Alternating intervals in which the RMSD is lower for one or the other reference structure indicate spontaneous transitions between Cluster 2-like and Cluster 1-like conformations during the simulation. These transitions demonstrate that the Cluster 1 geometry is dynamically accessible from a Cluster 2-like configuration within the gREST simulations.

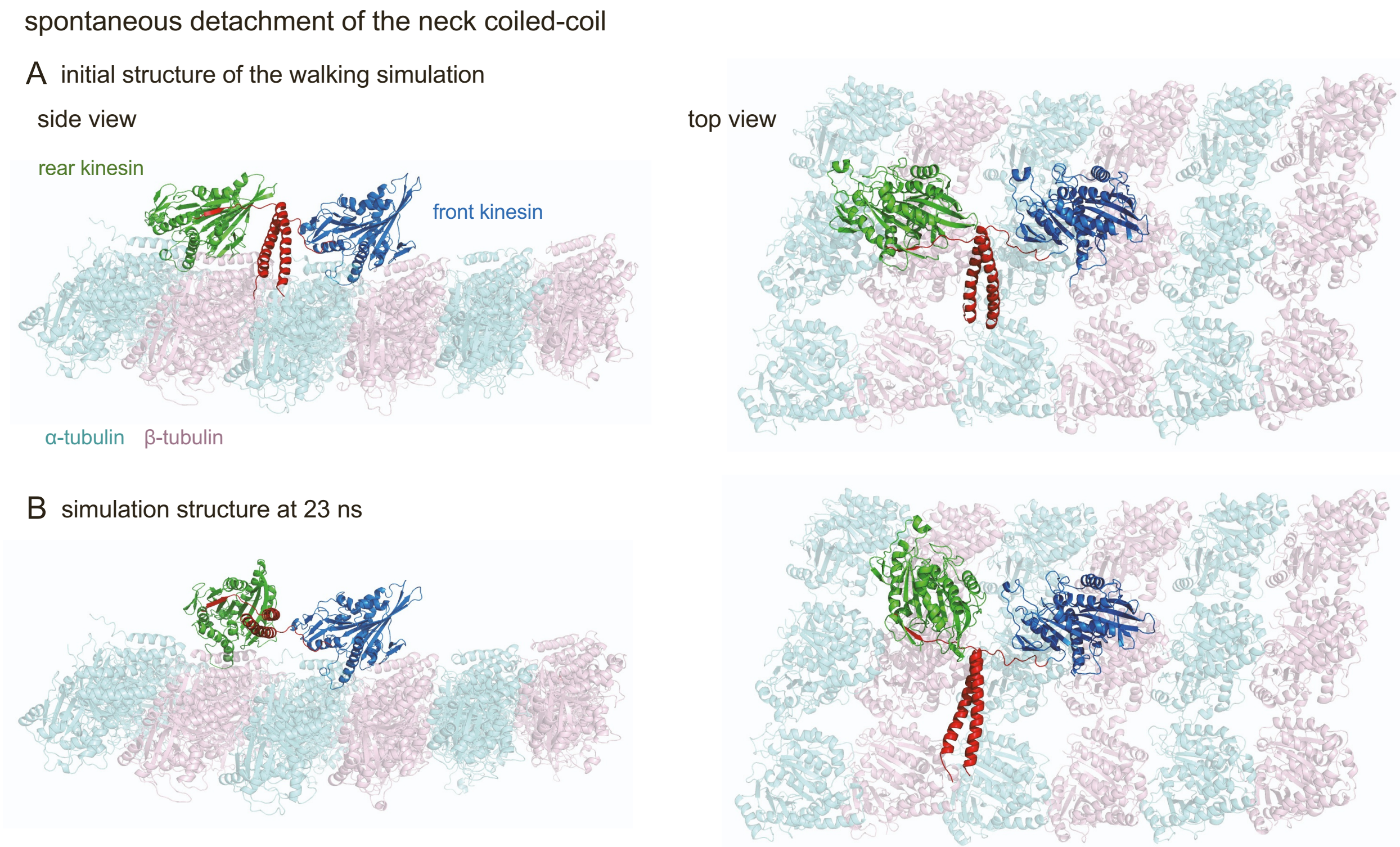

Figure S9: Spontaneous detachment of the neck coiled-coil from the microtubule observed in one of the initial stepping simulation trajectories. (A) Initial microtubule-bound conformation in which the neck coiled-coil is closely associated with the microtubule surface. To illustrate the full structural context, the $\alpha\beta$-tubulin subunits that were removed in the simulation setup have been reinserted. (B) A detachment event, in which the neck coiled-coil spontaneously separates from the microtubule.

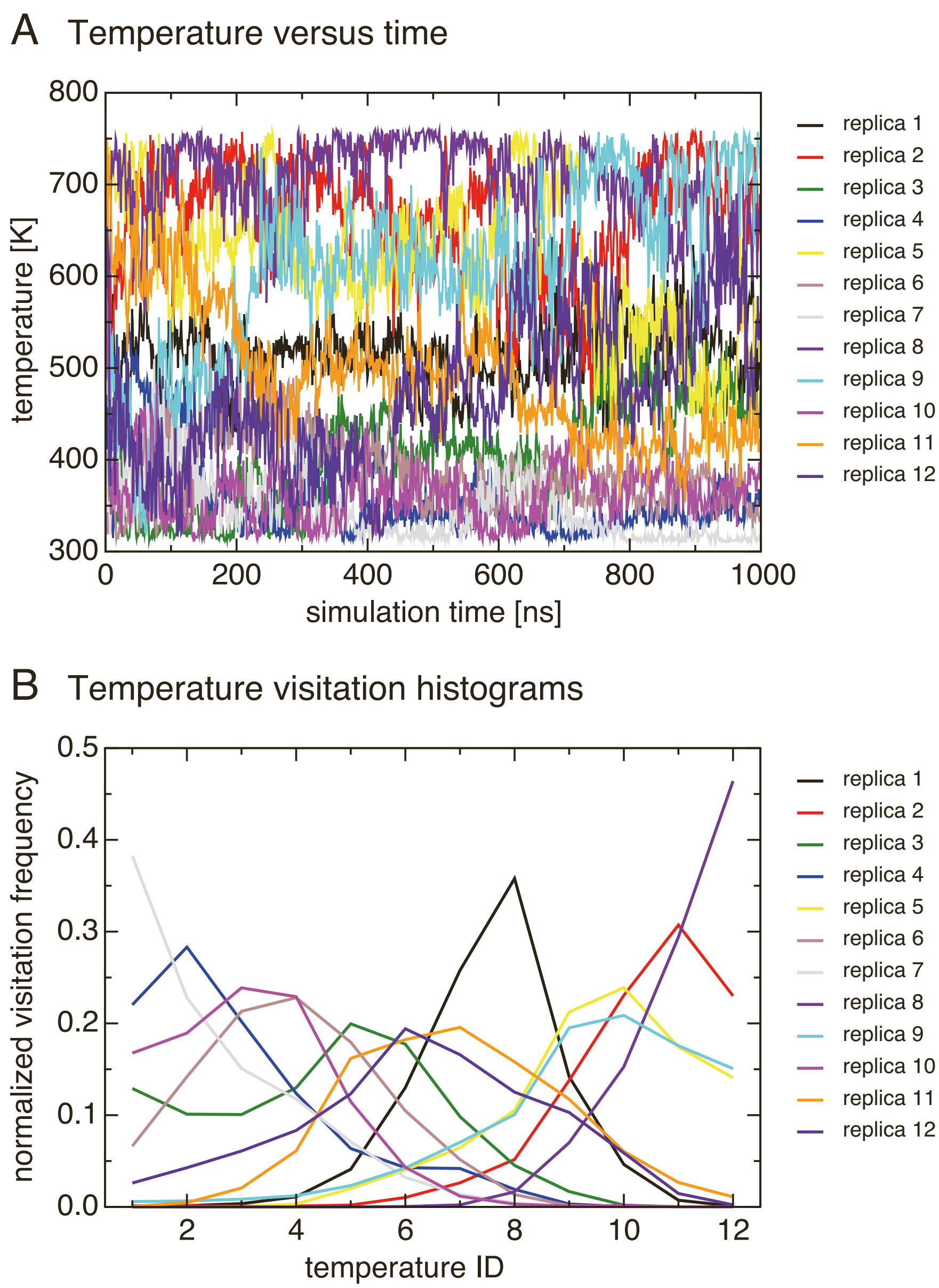

Figure S10: (A) Time evolution of the solute temperature for each replica in the gREST simulations. Replica-exchange attempts were performed every 10 ps; however, plotting the raw data at 10-ps intervals over the 1000-ns simulation results in an overly crowded representation. Therefore, the temperature data were divided into 1-ns blocks and averaged within each block. The resulting block-averaged temperatures (1000 data points per replica) are shown as a function of time, with colors corresponding to replica IDs 1–12. (B) Temperature-visitation histograms for all replicas in the gREST simulations. The horizontal axis indicates the solute-temperature ID (1–12), and the vertical axis shows the normalized visitation frequency. Each curve represents a single replica, with colors consistent with panel (A).

Table S1: Contact frequencies between the neck coiled-coil and the microtubule surface

kinesin[a]	tubulin[b]	frequency (%)[c]	kinesin[a]	tubulin[b]	frequency (%)[c]
**Cluster 1 ensemble**					
A:R355	E:D438	47.4	A:R355	E:S439	70.3
A:E362	E:K311	67.6	A:E362	E:Q342	33.3
A:R369	E:T340	47.4	A:R369	E:Q342	38.0
A:N370	Q:R156	38.8	A:N370	Q:V159	47.7
A:N370	Q:D160	37.4	B:K346	E:E433	48.7
B:K346	E:E434	34.7	B:E347	E:E433	34.0
B:E349	D:R390	94.9	B:K350	E:E433	53.2
B:K350	E:V437	39.0	B:K350	E:D438	33.0
B:K350	E:S439	33.5	B:K352	D:R390	52.7
B:I353	D:R390	98.0	B:I353	D:R391	80.4
B:I353	E:D345	31.9	B:I353	E:V437	46.9
B:L354	E:S439	53.9	B:N356	D:A387	34.4
B:N356	E:D345	47.7	B:T357	D:R391	30.1
B:T357	E:D345	69.7	B:W360	E:F343	38.6
B:W360	E:V344	30.7	B:W360	E:D345	75.0
B:R369	E:T337	34.6	B:R369	E:T340	43.1
B:R369	Q:D160	42.1	B:N370	E:K338	50.6
**Cluster 2 ensemble**					
B:K346	D:R390	32.7	B:E349	D:R390	46.6
B:E349	D:E412	31.6	B:R367	D:H307	30.1
B:R367	D:D427	38.8	B:R369	D:D427	38.5

Note: [a] Residues in the kinesin neck helix (rear: chain A; front: chain B). [b] α/β-tubulin chains located underneath the rear kinesin in the central protofilament are chains C/D, and those underneath the front kinesin are chains E/F. The corresponding α/β-tubulin chains in the adjacent protofilament on the right side when viewed toward the plus end are chains O/P and Q/R. [c] Only residue pairs with contact frequencies greater than 30% are listed.
